# Profile analysis and prediction of tissue-specific CpG island methylation classes

**DOI:** 10.1186/1471-2105-10-116

**Published:** 2009-04-21

**Authors:** Christopher Previti, Oscar Harari, Igor Zwir, Coral del Val

**Affiliations:** 1 Department of Molecular Biophysics, DKFZ, German Cancer Research Center, Heidelberg, Germany; 2 Department of Computer Science and Artificial Intelligence, CITIC-UGR (Research Center on Information and Comunication Technology), University of Granada, Granada, 18071, Spain; 3 Department of Molecular Microbiology, Howard Hughes Medical Institute, Washington University School of Medicine, St Louis, MO, USA; 4 Computational Biology Unit, Bergen Center for Computational Science, Sars Centre for Marine Molecular Biology, University of Bergen, Bergen, Norway

## Abstract

**Background:**

The computational prediction of DNA methylation has become an important topic in the recent years due to its role in the epigenetic control of normal and cancer-related processes. While previous prediction approaches focused merely on differences between methylated and unmethylated DNA sequences, recent experimental results have shown the presence of much more complex patterns of methylation across tissues and time in the human genome. These patterns are only partially described by a binary model of DNA methylation. In this work we propose a novel approach, based on profile analysis of tissue-specific methylation that uncovers significant differences in the sequences of CpG islands (CGIs) that predispose them to a tissue- specific methylation pattern.

**Results:**

We defined CGI methylation profiles that separate not only between constitutively methylated and unmethylated CGIs, but also identify CGIs showing a differential degree of methylation across tissues and cell-types or a lack of methylation exclusively in sperm. These profiles are clearly distinguished by a number of CGI attributes including their evolutionary conservation, their significance, as well as the evolutionary evidence of prior methylation. Additionally, we assess profile functionality with respect to the different compartments of protein coding genes and their possible use in the prediction of DNA methylation.

**Conclusion:**

Our approach provides new insights into the biological features that determine if a CGI has a functional role in the epigenetic control of gene expression and the features associated with CGI methylation susceptibility. Moreover, we show that the ability to predict CGI methylation is based primarily on the quality of the biological information used and the relationships uncovered between different sources of knowledge. The strategy presented here is able to predict, besides the constitutively methylated and unmethylated classes, two more tissue specific methylation classes conserving the accuracy provided by leading binary methylation classification methods.

## Background

The most important epigenetic modification of vertebrate DNA involves the addition of a methyl group to the carbon-5 of the pyrimidine ring of the cytosine in CpG dinucleotides (CpGs) [1-3]. The methylation of DNA provokes a localized restriction of transcription that can be used for the selective silencing of genes. This form of transcriptional control is mediated by regulatory regions termed CpG islands (CGIs) which overlap the promoter of all human housekeeping genes and over half of all tissue-specific genes [4-7]. CGIs are the only regions in the human genome that are rich in unmethylated CpGs [5] and therefore represent a notable exception to the almost "global" methylation that affects the bulk of the genome and has, over the time, resulted in the depletion of CpG dinucleotides from it.

The methylation of CGIs is associated with a host of normal and cancer-related processes [8-19], making them an important target for large-scale studies of DNA methylation that aim to shed light on their role in the epigenetic control of gene expression [20,21]. Nevertheless, measuring DNA methylation experimentally involves procedures that are time-consuming, expensive and have only recently been scaled to genome-wide approaches that maintain a high degree of resolution [3,22,23]. Computational solutions to the genome-wide prediction of CGI methylation would therefore be a great aid [24]. However, the characteristics that make a sequence susceptible or resistant to methylation are not completely understood.

Recent studies employing supervised machine learning methods account for differences between methylated and unmethylated DNA sequences [25-28]. However, recent experimental results have shown the presence of much more complex patterns of methylation in the human genome [29]. Since these patterns may vary across tissues and developmental stages, they are partially described by a binary methylation model [30]. Current computational methods therefore distinguish well between constitutively methylated and unmethylated CGIs, but do not take tissue-specific CGI methylation into account. This is a significant source of uncertainty, since their prediction models were trained on a heterogeneous mixture of constitutive and tissue-specific methylation. This could mask the characteristics that truly discriminate between CGIs that are methylated or unmethylated in all tissues. One of the primary causes for this situation was the lack of high-resolution methylation data from multiple healthy human tissues [25]. This impeded the discovery of tissue-specific CGI methylation classes and the key characteristics that predispose certain CGI sequences to either constitutive or tissue-specific methylation.

The data of Human Epigenome Project (HEP) [31] gives us the opportunity to try and resolve this issue. They specify the methylation status of more than 30000 individual CpGs from the human chromosomes 6, 20 and 22 in twelve healthy tissues and cell types, therefore representing the highest-quality source of experimental methylation data currently available [31] and have been used before to gain insights into the epigenetic variability of the human genome [29]. In this paper we use this information to identify novel profiles that map DNA sequence, structural, physicochemical and evolutionary attributes of CGIs into methylation profiles. They clearly distinguish CGIs that are constitutively methylated or unmethylated from CGIs that show a tissue-specific degree of methylation. At the same time, these profiles provide important insights into the key attributes that determine if a CGI has a functional role in the epigenetic control of gene expression and is predisposed to become methylated during normal cellular differentiation.

## Results and discussion

In recent years, there have been several successful efforts at predicting the methylation status of CGIs. These methods use different combinations of DNA patterns and attributes to classify them as methylated or unmethylated [25-27,32], but do not take tissue-specific CGI methylation into account. In this work we elucidate intermediate subclasses of CGIs with a differential degree of methylation that better reflect the complex methylation patterns of the human genome.

**Figure 1:**
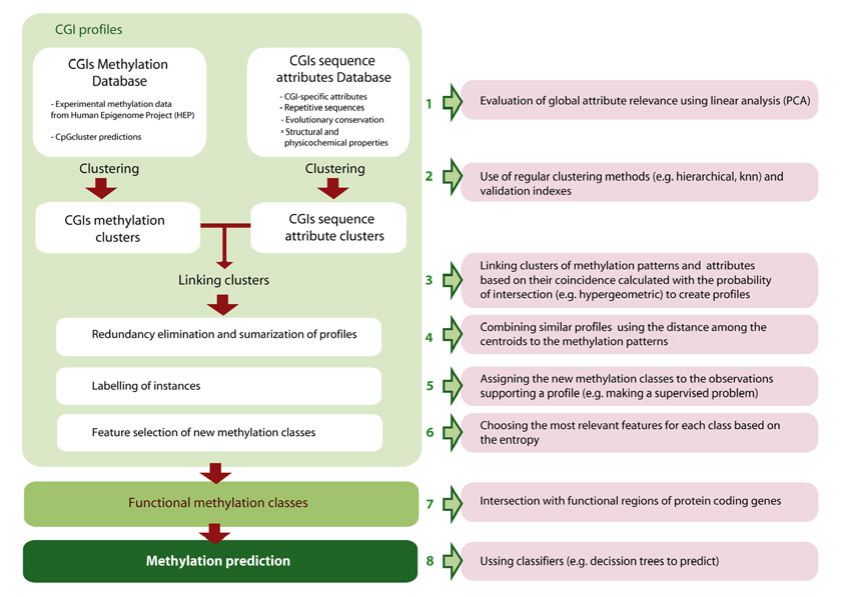
**Overview of the profile-based approach to the analysis of tissue-specific CGI methylation**.

Our approach is primarily a mining process carried out in the absence of supervised data [33-37] (Fig. 1) that identifies associations among two different data domains and uses these associations to label the database. First, we independently clusterer two different data-domains: CGI methylation and sequence-related attributes. Then, both domain-clusters are related and evaluated based on the probability of intersection using the hypergeometric measurement. This measure helps to uncover cohesive relational clusters while avoiding conditional decisions -often derived from the use of conditional probabilities–of clustering first one domain and then the other or viceversa [34].

Once the database is labeled, after a summarization process, we proceed with the corresponding feature selection of the new methylation classes and inference steps with the creation of a classifier with the newfound knowledge.

### 1. Profile identification

We selected CGIs that were covered to over 70% by the CpGs in the HEP dataset, requiring their methylation status to be defined in at least 2 tissues. This measure ensured a balanced dataset, where more than two-thirds of the CGIs were defined in all tissues and 493 (95%, of the CGIs) were defined in at least 10 tissues (Table 1, Additional file 1).

**Table 1:** Unique CpGs and CGIs defined by the HEP data

Tissue/Cell type	Abbreviation	Total # CpGs (%)*	# CGIs (%)**
CD4 T lymphocytes	CD4	31,219 (94.86)	515 (99.61)
CD8 T lymphocytes	CD8	29,979 (91.09)	503 (97.29)
Dermal fibroblasts	DF	29,776 (90.48)	504 (97.49)
Dermal keratinocytes	DK	29,739 (90.36)	508 (98.26)
Dermal melanocytes	DM	29,809 (90.58)	504 (97.49)
Fetal liver	FL	24,343 (73.97)	452 (87.43)
Fetal skeletal muscle	FSM	24,250 (73.69)	448 (86.65)
Heart muscle	HM	31,268 (95.01)	517 (100)
Liver	-	31,456 (95.58)	517 (100)
Placenta	-	29,900 (90.85)	505 (97.68)
Skeletal muscle	SM	31,518 (95.77)	513 (99.23)
Sperm	-	23,621 (71.77)	444 (85.88)

Supported by at least 2 tissues:		32,910	517

* Number and percentage of CpGs measured in at least one of the twelve tissues.

** Number and percentage of CGIs where at least 70% of the CpGs were covered by the HEP data, in at least two tissues.

The CGIs were identified by the *CpGcluster *algorithm [38], which does not rely on the traditional three parameters of length, GC-content and O/E-ratio [39]. Instead, it searches for closely spaced CpGs and computes the probability of finding a cluster with the same length and number of CpGs in the genome (*p-value*). *CpGcluster *has a high degree of sensitivity for detecting known, functional CGIs, while at the same time, excludes spurious repetitive elements [38]. Over 97% of the CGIs in our dataset were covered by a CGI predicted using the traditional parameters [39], while the inverse ratio is 71%. This lower degree of coverage is due the high specificity of CpGcluster that excludes more false-positives, such as *Alu*-repeats, than methods based on the traditional thresholds.

We then characterized each of the CGIs in our dataset using 38 attributes belonging to four distinct categories: (1) CGI-specific attributes (e.g. their G+C content, Observed/Expected ratio and CpGcluster *p*-value), (2) Repetitive sequences (number and type of repetitive elements); (3) Evolutionary conservation (e.g. *PhastCon *content), as well as (4) Structural and physicochemical properties of the DNA itself (e.g. twist, tilt, roll, shift, slide and rise) [40]. The attribute global linear analysis (PCA analysis) (Additional file 2) showed that all of them contributed significantly to the overall variability of the dataset. Therefore, all raw data were used in further steps because they provide a more interpretable characterization.

Then attribute and methylation data were independently clustered by both hierarchical and k-means clustering methods. The validity indices that define the appropriate number of clusters were not conclusive, as shown in Table 2. Thus we selected cluster partitions yielding more than two clusters using the best two partition scores (see Methods).

**Table 2:** Validity indices used to estimate the optimum number of data clusters

CGI methylation data			
Hierarchical clusters	ICT	K-means clusters	ADSM

**9**	**2.35**	**3**	**0.755**
**16**	**2.3**	**4**	**0.678**
35	2.25	5	0.605
38	2.2	6	0.509
41	2.15	7	0.497
44	2.1	8	0.490
48	2.05	9	0.472
50	2	10	0.485

**CGI attribute data**			

Hierarchical clusters	ICT	K-means clusters	ADSM

1	2.7	**3**	**0.221**
**8**	**2.65**	**4**	**0.233**
**9**	**2.6**	5	0.184
9	2.55	6	0.177
9	2.5	7	0.169
16	2.45	8	0.145
24	2.4	9	0.134
31	2.35	10	-0.348

**Figure 2:**
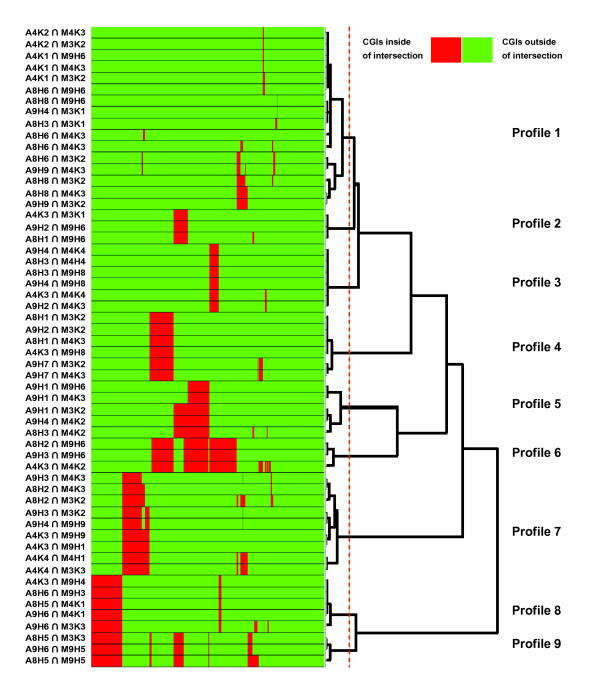
**Determining non-redundant CGI profiles**. Elimination of redundant CGI profiles. Initially, 55 profiles (relations between CGI sequence attributes and methylation classes linked by the probability of intersection) were identified. We grouped all profiles recognizing the same observation using a column/row hierarchical clustering, and summarize each cluster by their most representative prototype (i.e., the most supported relation of each cluster). The validity index we used (see methods) suggests a partition into 9 final profiles.

In order to determine a combination of biological CGI attributes that naturally intersected with a specific pattern of methylation, we linked the two pairs of clusters by calculating the probability of intersection (*PI*) and employing a significance *p*-value < 0.05. This approach optimizes the cluster partitions based on the coincidence between independent clusters [37] instead of intrinsic intra/inter clustering measurements [41]. The application of this unsupervised process to our dataset identified 55 significant intersections (profiles) where two independent clusters had more CGIs in common than would be expected by chance (Additional file 3). These 55 profiles are redundant due to the fact that partitions from distinct numbers of clusters were allowed in the former step. Therefore, a cluster from one domain might be related to more than one cluster from the other domain and vice versa. We removed this redundancy (Figure 2a) by grouping the 55 profiles and selecting a representative prototype from those that recognize similar observations. The process resulted in 9 non-redundant profiles (PBC) (Figure 2), which demonstrate clear patterns of tissue-specific methylation (Table 3) associated with distinct biological characteristics (Table 4). The attribute values in Table 4 were normalized between 0 and 1. This normalization is performed before the clustering process in order to prevent bias clusters caused by attributes with high absolute values. The significance at a *p*-value < 0.05 is relative to these normalized values. The non-normalized values are available in the supplementary information. The number of CGIs recovered with each profile is registered in Table 5.

**Table 3:** CGI profiles: Methylation values of each non-redundant CGI profile.

	Constitutively methylated	Unmethylated in sperm	Differentially methylated	Constitutively unmethylated					
**Tissue/Cell type**	**1**	**2**	**3**	**4**	**5**	**6**	**7**	**8**	**9**

CD4	**0.916**	**0.881**	**0.655**	0.074	0.078	0.065	0.076	0.094	0.072
CD8	**0.912**	0.905	**0.683**	0.089	0.078	0.071	0.104	0.136	0.092
DF	**0.876**	**0.758**	**0.595**	0.070	0.066	0.068	0.079	0.082	0.045
DK	**0.866**	**0.708**	**0.531**	0.077	0.061	0.063	0.072	0.074	0.086
DM	**0.900**	0.854	**0.734**	0.093	0.103	0.079	0.102	0.133	0.108
FL	**0.842**	0.762	**0.562**	0.104	0.117	0.105	0.138	0.158	0.157
FSM	**0.842**	0.793	**0.619**	0.097	0.136	0.127	0.125	0.118	0.052
HM	**0.913**	**0.867**	**0.688**	0.068	0.071	0.059	0.064	0.072	0.059
Liver	**0.900**	0.883	**0.691**	0.073	0.071	0.065	0.080	0.095	0.077
Placenta	**0.878**	0.846	**0.679**	0.085	0.075	0.066	0.096	0.098	0.123
SM	**0.866**	0.804	**0.558**	0.071	0.072	0.057	0.062	0.078	0.067
Sperm	**0.836**	**0.178**	**0.359**	0.102	0.086	0.105	0.125	0.117	0.112

Average methylation ± Std	**0.879 ± 0.065**	**0.770 ± 0.1**	**0.613 ± 0.202**	0.084 ± 0.066	0.085 ± 0.068	0.078 ± 0.058	0.094 ± 0.074	0.105 ± 0.088	0.088 ± 0.065

Average and tissue-specific methylation values of the nine non-redundant CGI profiles. The methylation values mentioned during the comparison of the profiles are marked in bold and all differences between profiles are supported by a MWW *p*-value lower than 0.01.

**Table 4:** CGI profiles: Attribute values of each non-redundant CGI profile.

	Constitutively methylated	Unmethylated in sperm	Differentially methylated	Constitutively unmethylated					
Attributes	**1**	**2**	**3**	**4**	**5**	**6**	**7**	**8**	**9**

*CpGcluster p*-value^1^	**0.112**	**0.130**	**0.229**	**0.047**	**0.020**	**0.024**	**0.042**	**0.033**	**0.053**
O/E ratio^1^	0.239	0.253	**0.380**	**0.263**	**0.255**	**0.241**	**0.239**	**0.208**	**0.269**
CpG distance^1^	**0.468**	**0.439**	**0.345**	**0.510**	**0.419**	**0.382**	**0.353**	**0.374**	**0.286**
SD^1^	**0.506**	**0.518**	**0.501**	**0.578**	**0.578**	**0.616**	**0.594**	**0.556**	**0.625**
G+C content^1^	**0.336**	**0.347**	**0.438**	**0.259**	**0.335**	**0.405**	**0.474**	**0.481**	**0.567**
Repetitive content^2^	0.010	0.000	0.002	0.006	0.005	0.017	0.036	0.042	**0.119**
Repetitive elements^2^	0.004	0.000	0.004	0.012	0.004	0.037	0.066	0.078	**0.192**
*PhastCon *content^3^	**0.722**	**0.700**	**0.521**	0.063	0.057	**0.102**	**0.101**	0.081	0.092
*PhastCon *elements^3^	0.330	0.336	0.242	0.049	0.060	0.076	0.064	0.066	0.050
CG^4^	0.097	0.101	**0.222**	**0.082**	**0.104**	**0.120**	**0.138**	**0.130**	**0.179**
GC^4^	0.203	0.215	**0.267**	**0.149**	**0.195**	**0.245**	**0.255**	**0.230**	**0.318**
AA^4^	0.171	0.156	0.141	**0.342**	**0.211**	**0.172**	**0.130**	**0.118**	**0.088**
TT^4^	0.181	0.239	0.154	**0.327**	**0.352**	**0.212**	**0.160**	**0.158**	**0.162**
TA^4^	0.325	0.350	0.212	0.323	0.225	0.194	0.160	0.180	0.116
AT^4^	0.247	0.305	0.211	**0.404**	**0.303**	**0.221**	**0.149**	**0.119**	0.161
CA^4^	**0.485**	**0.391**	**0.363**	0.407	0.320	0.360	0.340	0.358	0.208
TG^4^	**0.540**	**0.525**	**0.383**	0.386	0.446	0.453	0.415	0.375	0.460
AC^4^	0.465	0.351	0.399	0.477	0.361	0.379	0.340	0.305	0.193
GT^4^	0.446	0.410	0.456	0.391	0.420	0.338	0.329	0.363	0.329
AG^4^	0.467	0.447	0.360	0.498	0.413	0.465	0.448	0.401	0.360
CT^4^	0.463	0.493	0.443	0.437	0.532	0.492	0.436	0.445	0.401
CC^4^	0.303	0.323	0.277	**0.303**	**0.330**	**0.337**	**0.384**	**0.469**	**0.324**
GG^4^	0.296	0.322	0.292	**0.311**	**0.339**	**0.366**	**0.423**	**0.394**	**0.602**
GA^4^	0.381	0.359	0.306	0.429	0.336	0.376	0.362	0.298	0.270
TC^4^	0.352	0.355	0.417	0.350	0.393	0.314	0.299	0.378	0.213
Bending^5^	0.597	0.596	**0.567**	**0.376**	**0.507**	**0.639**	**0.695**	**0.699**	**0.789**
Curvature^5^	0.506	0.547	**0.410**	**0.669**	**0.712**	**0.653**	**0.545**	**0.561**	**0.405**
Stacking energy^5^	**0.771**	**0.776**	**0.671**	**0.840**	**0.790**	**0.739**	**0.711**	**0.721**	**0.666**
Turns^5^	0.923	0.920	**0.799**	**0.938**	**0.943**	**0.942**	**0.925**	**0.928**	**0.908**
Degree of twist^5^	0.920	0.916	**0.794**	0.936	0.940	0.940	0.921	0.925	0.903
DNA cleavage^5^	0.192	0.187	**0.234**	**0.139**	**0.160**	**0.191**	**0.209**	**0.189**	**0.285**
Bases per turn^5^	0.074	0.078	**0.195**	0.060	0.055	0.057	0.073	0.070	0.090
Twist constraint^5^	0.763	0.749	**0.635**	**0.837**	**0.780**	**0.768**	**0.736**	**0.724**	**0.654**
Tilt constraint^5^	0.645	0.657	0.565	0.714	0.729	0.725	0.738	0.768	0.683
Roll constraint^5^	0.484	0.504	0.467	0.453	0.522	0.572	0.547	0.471	0.652
Shift constraint^5^	0.606	0.592	0.527	0.721	0.683	0.672	0.674	0.696	0.587
Slide constraint^5^	0.611	0.606	0.677	0.678	0.652	0.645	0.578	0.469	0.662
Rise constraint^5^	0.536	0.571	0.466	0.548	0.617	0.649	0.631	0.583	0.691

The attribute values in Table 4 are normalized between 0 and 1. This normalization is performed before the clustering process in order to prevent biased clusters caused by attributes with high absolute values. The significance at a p < 0.05 is relative to these normalized values. The non-normalized values are available in the supplementary information. The attribute values mentioned during the comparison of the profiles are marked in bold and all differences between profiles are supported by a MWW p-value lower than 0.01.

^1^CGI-specific attributes; ^2^Repetitive sequences; ^3^Evolutionary conservation; ^4^Dinucleotide content; ^5^Structural and physicochemical properties;

**Table 5:** Profile support

Profile	# CGIs (%)	Methylation pattern
1	84 (16.25)	Constitutively methylation
2	50 (8.51)	Unmethylated in sperm
3	36 (6.69)	Differentially methylated
4	68 (13.15)	Constitutively unmethylated
5	36 (6.96)	
6	62 (11.99)	
7	76 (14.70)	
8	60 (11.60)	
9	29 (5.61)	

Misclassified	22 (4.26)	

A CGI was defined as being misclassified if it lacked an experimental methylation value in sperm but was classified nonetheless as constitutively methylated or unmethylated solely in sperm. CGIs that were missing methylation data in more than half of tissues and cell types under analysis were also defined as misclassified and removed.

**Figure 3:**
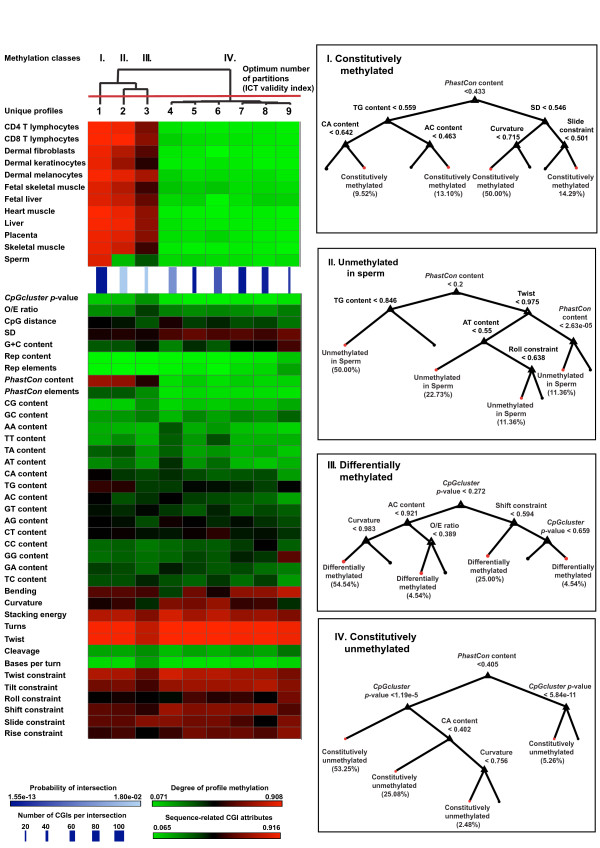
**Linking clusters and Feature selection of new methylation classes**. Summarization and feature selection of CGI profiles. A) Identification of 9 CGI profiles by linking CGI sequence attribute clusters (lower left corner) and methylation clusters (upper left corner) by the probability of the intersection (PI), which is calculated based on the hypergeometric measurement (blue color). The attributes were normalized within the colourmap intervals. Notably, the relations are built based on the *PI *(line color; dark blue: low *p*-value; light blue: high *p*-value), which substantially differs from the typical support of intersection measurement (line weight; thin: few; tick: many). For example, the fifth relation (5th column from left) is supported by just ~40 observations (thin line) but most of the CGI sequence attribute observations correspond to the 4th methylation class and only few belong to others classes. This approach can generate cohesive relations even if they aren't highly supported. The nine methylation profiles are summarized by similarity of their prototypes, constituting 4 final methylation classes (I-IV). These classes were used to label all CGI sequence attributes observations. B) Feature selection for each class based on the dataset labeled in A). This process has been carried out locally by using decision trees (*Matlab*) where the desired class (labeled read leaf) was distinguished from all of the others (unlabeled black leaf).

Finally, we labeled the observations using the corresponding methylation patterns (Figure 3a, Figure 3b) into four methylation classes: *Constitutively methylated*, containing CGIs that are highly methylated in all tissues (Profile 1); *Unmethylated in sperm *contained CGIs that only lacked methylation in sperm (Profile 2); *Differentially methylated *contained CGIs that showed a distinct degree of methylation for each tissue (Profile 3); and *Constitutively unmethylated*, comprising CGIs that are uniformly unmethylated across all tissues and cell types (Profiles 4 through 9).

The initial PCA analysis suggested that all attributes were informative. However, we considered the data labeled in the previous step and applied a local feature selection analysis based on the entropy of the attributes (i.e. decision tree) for dissecting each new methylation class. The most relevant attributes were the novel ones included in this study: *PhastCon *content, the *p*-value computed by the *CpGcluster *algorithm, and structural characteristics of the CGIs describing their three-dimensional flexibility such as: twist, tilt, roll, shift, slide and rise of a DNA sequence from a novel model of dinucleotide stiffness. Moreover, a correlation analysis of all attributes shows that each relevant attribute was not replaceable by any other (Additional file 6). Here we describe the most relevant attributes for each class.

#### Constitutively methylated class

CGIs in this class had a high average degree of methylation in all tissues (avg_meth > 0.8), and reflect PBC1. This class is described by a higher average content of CA and TG dinucleotides, which can be seen as the "footprint" of methylation, since they are often the result of the deamination of methylated cytosine. The most notable difference between the constitutively methylated class and the other classes, specially the constitutively unmethylated, is its higher overlap with *PhastCon *elements. This attribute never reaches values greater than 0.1 in the former class, while it never falls below 0.5 in the differentially methylated classes. A high *PhastCon *overlap was originally seen as a sign of a potential conserved functional regulatory element [38,42] however their high degree of methylation poses limits on their functionality by restricting access to the DNA.

#### Constitutively unmethylated class

The CGIs in this class have a low avg_meth (≤ 0.2) in all tissues; it summarizes *PBCs *from *4 *through *9*. Previous results have shown a negative correlation between the concentration of CpGs and a high degree of methylation, and this idea has been used as the starting point for methylation prediction. However, our findings agree with the new experimental work of Raykan et al. [43]. The authors find that DNA methylation can occur at high-, medium- and, contrary to previous notions, at even some low-CpG density promoters. It has been also found that certain promoters with few CpGs were active and methylated, whereas other promoters of that group can be unmethylated when active [44]. These data suggest that DNA methylation is involved in regulating activity over a broad range of CpG O/E-ratios, including CpG-poor promoters, located in tissue-specific differentially methylated regions (tDMRs).

All constitutively unmethylated PBCs are very similar, with exception of the unexpectedly high content in GC-rich repetitive elements of PBC 9. Only 6% of the CGIs supported this PBC indicating that the combination of attributes learned from the few remaining repetitive elements is not representative of a large subset of CGIs. This is a consequence of the HEP selection process, which excluded most of these repeats [31]. However, in our results, 36 out of the 46 CpG islands overlapping with a repetitive element, overlap either with the extended promoter region (14 CpG islands) or with the TSS of a gene (22 CGIs). Of these CGIs, 32 (70%) are unmethylated despite the presence of a repeat. In fact, all TSS-overlapping CGIs are unmethylated regardless of the repeat in their vicinity. The presence of a promoter seems to be incompatible with the methylation of these repeats. This small set agrees with the recent experimental findings of Meissner et al. [30], where it was proven that not all transposable elements are equally affected concerning methylation. Long interspersed nuclear elements (LINEs) and long terminal repeats (LTR) are generally methylated independently of CpG density. However, CpG density influences whether non-autonomous short interspersed nuclear elements (SINEs) and low complexity regions remain unmethylated, which may be a reason for the low degree of methylation of the repetitive elements of profile 9. These results show that CGIs with distinct biological characteristics can share the same methylation status (Figure 2a) and that the degree of CpG enrichment or the presence of a repetitive element alone does not determine if a sequence is protected against methylation.

These results show that CGIs with distinct biological characteristics can share the same methylation status (Figure 2a) and that the degree of CpG enrichment or the presence of a repetitive element alone does not determine if a sequence is protected against methylation.

The *CpGcluster p*-value is a key attribute for distinguishing between constitutively methylated and unmethylated CGIs. This measure distinguishes true CGIs from repetitive *Alu*-elements, which are the main source of false-positive CGI predictions, and is not linked to either G+C content or the O/E ratio. This is important because it can determine the significance of a CGI independently of changes in the G+C content of the genomic sequence and is therefore not affected by fluctuations in the sequence composition. G and C-containing dinucleotides (CpC, CpG, GpG) in conjunction with a reduction in A and T-containing dinucleotides (ApA, TpT) and the curvature are also important attributes for this class. The high contents of G and C dinucleotides leads to an increasing degree of sequence bending and reduces both the macroscopic curvature of the DNA as well as the amount of energy needed to separate the strands (stacking energy). Despite the increasing G+C content, the O/E ratio decreases continuously (Figure 2c), indicates that there is a balance between CpG enrichment on one hand and the overall G+C content on the other.

In addition to the previously known classes we found two tissue-specific methylation classes:

#### Unmethylated in sperm class

These CGIs lack methylation in sperm, and have a slightly lower degree of methylation in CD4 T lymphocytes, dermal fibroblasts, dermal keratinocytes and the muscle tissue of the heart. Nevertheless, sperm is the only tissue that shows a level of methylation below 0.2 making it the defining characteristic of this class. This agrees with the known normal development and control of sperm-specific gene expression of germ cells [45], as well as with their epigenetic reprogramming during gametogenesis.

#### Differentially methylated class

In contrast to the classes constitutively methylated and unmethylated in sperm, this class showed a heterogeneous degree of methylation ranging across all tissues. It presented both highly methylated and unmethylated CGIs in the same tissue yielding an intermediate degree of methylation after averaging. This average was significantly lower than that of the CGIs unmethylated in Sperm. This class also presented the lowest average distance between CpGs, the highest CpG O/E value, G+C content, and CpG and GpC dinucleotide content than the constitutively methylated and unmethylated in sperm classes. This class presented unique structural characteristics, such as low degrees of bending and curvature, as well as a high degree of solvent-accessibility of the DNA backbone (*DNA cleavage*), which may indicate a higher degree of permissiveness for DNA binding. DNA sequence bending is generally higher if the sequence contains phased GGGCCC sequences, and therefore the bending should be higher for sequences rich in G+C. This does not apply to this class where alternating CpGs and GpCs limit bending, curvature, twist and the number of helix turns, but tends to increase the flexibility of the sequence.

### 2. Functional CGI categorization by gene association

In order to assess the functionality of the four newly defined methylation classes, we measured the coincidence between them and defined gene association classes (Table 6) using probability of intersection (*PI*) (Table 7). The *PI *is usually employed to perform coincidence analysis because it is a context-sensitive metric that takes into account the domain where the intersection is calculated. It measures the degree of inclusion of one set into another, considering both the number of instances intersected between two sets as well as those instances not belonging to the intersection. Formally, the *PI *determinates the *p*-value, which is the statistical significance of observing over represented intersected instances (i.e. occur more frequently than could be expected by pure chance). The results obtained show that the new classes represent unique, functional CGI profiles associated with distinct CGI methylation patterns and gene compartments (Table 8).

**Table 6:** Distribution of CGIs over the gene association classes

Gene association class	Location of CGI	# CGI (%)
Pseudogene	Within 1.5 kb of a pseudogene	62 (12.53)
TSS	Overlapping TSS	146 (29.49)
Promoter	Overlapping extended promoter region, (1.5 kb upstream of the TSS to end of the 5'UTR)	178 (35.96)
3'UTR	Overlapping 3' UTR and may overlap CDS	12 (2.42)
CDS	Overlapping protein coding region	51 (10.30)
Intron	Lies entirely within an Intron (excluding 3' and 5' UTRs)	29 (5.86)
NA	Outside of the gene environment	17 (3.43)

**Table 7:** Coincidence between gene association classes and PBCs

Gene association class	Constitutively unmethylated (*PI*)	Constitutively methylated (*PI*)	Unmethylated in sperm (*PI*)	Differentially methylated (*PI*)
TSS	**138** **(5.27E-13)**	3	3	2
Promoter	118	28	17	15
3'UTR	1	**6 (1.2E-03)**	1	4
CDS	14	**23 (9.29E-08)**	7	7
Intron	12	8	0	**9 (7.3E-05)**
Pseudogene	33	11	**14 (7.6E-05)**	4
NA	7	5	2	3

Sum (% of total)	323 (65.25)	84 (16.97)	44 (8.89)	44 (8.89)

Only the *p-value *(*PI *of significant intersections (< 0.01) is shown and marked in bold.

**Table 8:** Re-classification using functional CGI profiles

Methylation class	# CGI (%)	Significant gene associations	*PI*
Constitutively unmethylated	323 (65.25)	Promoter/TSS	1.00E-12
Constitutively methylated	84 (16.97)	CDS	4.27E-08
Unmethylated in sperm	44 (8.89)	Pseudogenes	3.5E-03
Differentially methylated	44 (8.89)	Introns	4.01E-04

The CGIs in our dataset were associated with 497 protein-coding gene loci (Table 6). While the vast majority of the CGIs (> 68%) were located in the vicinity of a promoter, less than 4% of the CGIs were outside the genic environment, indicating that the dataset is skewed towards genic regions. This is a consequence of a bias in the HEP data, which includes few intergenic regions [31]. Normally a higher percentage of CGIs would be found outside of the genic region in an unbiased dataset [38]. Approximately 20% of the CGIs are located in the gene-body and we found that these CGIs were unequally distributed, since there were approximately 30% more CDS-overlapping CGIs than those located in introns.

Moreover, it is known that CGIs associated with the gene-body are susceptible to both constitutive as well as tissue-specific methylation [2,43,46]. However, by separating between coding and non-coding regions, we were able to distinguish between highly methylated and differentially methylated CGIs.

#### Constitutively methylated class

It coincided significantly with the CDS of the genes in our dataset. They are highly conserved and may be the result of GC-rich codons simulating the presence of a CGI. The methylation of a CDS region itself, in contrast to a TSS region, does not impede the progression of transcription, making this region permissive for both methylation and compact chromatin conformation.

#### Differentially methylated class

This class coincided significantly with CGIs located in introns, indicating the presence of functional methylation-dependent sequence elements. Though the majority of the differentially methylated CGIs that were conserved overlapped with the CDS (Table 9), we found that they were the only class of CGI that was significantly enriched in highly conserved non-coding elements (HCNEs) [47] (Additional file 5). Since the differential methylation of HCNEs has recently been shown in a comparison of embryonic stem cells (ES) and ES-derived differentiated cells in mouse [30] these differentially methylated CGIs may represent examples of enhancers that are controlled by methylation [48,49].

**Table 9:** Distribution of conserved and not conserved CGIs over PBCs and gene association classes

Conserved CGIs				
**Gene association class**	Constitutively unmethylated (%)*	**Constitutively methylated (%)**	**Unmethylated in sperm (%)**	**Differentially methylated (%)**

TSS	75 (23.22)	2 (2.38)	-	-
Promoter	43 (13.31)	24 (28.57)	8 (18.18)	7 (15.91)
3'UTR	1 (0.31)	2 (2.38)	1 (2.27)	3 (6.82)
CDS	12 (3.72)	23 (27.38)	7 (15.91)	7 (15.91)
Intron	6 (1.86)	3 (3.57)	-	2 (4.55)
Pseudogene	18 (5.57)	8 (9.52)	7 (15.91)	2 (4.55)
NA	2 (0.62)	1 (1.19)	2 (4.55)	3 (6.82)
Total # of conserved CGIs	157 (48.61)	63 (75.00)	25 (56.82)	24 (54.55)

**Not conserved CGIs**				

**Gene association class**	Constitutively unmethylated (%)*	**Constitutively methylated (%)**	**Unmethylated in sperm (%)**	**Differentially methylated (%)**

TSS	63 (19.50)	1 (1.19)	3 (6.82)	2 (4.55)
Promoter	75 (23.22)	4 (4.76)	9 (20.45)	8 (18.18)
3'UTR	-	4 (4.76)	-	1 (2.27)
CDS	2 (0.62)	-	-	-
Intron	6 (1.86)	5 (5.95)	-	7 (15.91)
Pseudogene	15 (4.64)	3 (3.57)	7 (15.91)	2 (4.55)
NA	5 (1.55)	4 (4.76)	-	-
Total # of not conserved CGIs	166 (51.39)	21 (25.00)	19 (43.18)	20 (45.45)

Total # CGIs per PBC	323	84	44	44

*Absolute number and percentage of conserved and not conserved CGIs of each PBC in the gene-association classes.

#### Unmethylated in sperm class

Over 40% of the CGIs belonging to this class coincided with promoter-overlapping CGIs, including genes known to be testis-specific, such as DDX43 [16], HIST1H2BA [50], PIWIL3 [51] and ECAT1 [52]. Although this supports the view that germline-specific genes are preferentially methylated in somatic tissues [44], the only significant intersection with a gene-class was with the pseudogene-proximal CGIs, 22% of which were unmethylated in sperm. Only 12% of all CGIs were associated with pseudogenes and the majority of them represent "processed" pseudogenes (> 64%). This may still include parts of the core promoter region, including the promoter-overlapping CGI [53]. Their lack of methylation in sperm was thought to be a by-product of the global changes in methylation that occur during spermatogenesis [45]. Although it may also permit them to be transcriptionally active in sperm [44] they are normally targeted for silencing through methylation during differentiation and therefore show a high degree of methylation in somatic tissues [45]. This complicates the identification of CGIs that are involved in controlling the sperm-specific expression of protein-coding genes via promoter-CGI methylation such as the MAGE and HAGE-genes [9,54] because it may lead to false positives in genome-wide studies of promoter methylation. For example, DPPA5 a functional testis-specific gene [55], is active in pluripotent cells and down-regulated during the differentiation process [56], but we found that it contains a CGI and a pseudogene within its 5'UTR. Therefore it is not clear if the lack of methylation of this CGI is necessary to maintain tissue-specific activity or simply a by-product of the pseudogene in its vicinity.

#### Constitutively unmethylated class

This class showed significant coincidence with the CGIs overlapping the TSS. However, they showed neither the highest G+C-content nor the highest O/E ratio of the whole unmethylated group. Instead, they showed the lowest average *CpGcluster p*-value, further supporting the use of this attribute as a better measure of functionality than the CpG enrichment or G+C content alone [38].

This categorization of the methylation classes was used to re-classify the dataset into the four functional methylation classes shown in Table 8. The promoter/TSS proximal CGIs represent the vast majority of all CGIs and they are predominantly unmethylated. It has been estimated that about 18% of the CGIs in the human genome are subject to tissue-specific methylation [43] and we found our data to support this estimate since just over 17% of the CGIs were either unmethylated in sperm or differentially methylated. It is noteworthy to mention that neither of these two classes overlapped significantly with the promoter-proximal CGIs.

### 3. Prediction of CGI methylation

Our four functional CGI methylation classes were then employed in the development of a supervised classifier. Our hypothesis was that if these classes were biologically and computationally significant, they would be useful in predicting new observations. To do so we first labeled each observation using the classes assigned by the profiles. We then used a simple classifier (i.e. decision tree) that employs 23 of the 38 attributes to predict the four classes of methylation. The methods were tested via 10-fold cross-validation where imbalanced classes were compensated [57] (see Methods).

These results show that the decision tree can encode rules that predict CGIs with distinct methylation patterns at a high level of accuracy (Table 9, Additional file 4). It is difficult to asses the performance of our method compared to previous computational approaches due to the fact that all constrain the prediction to only two methylation classes, where a sequence is either "methylated" or "unmethylated" across all possible tissues or cell types [26,32] and they do not take into account tissue-specific methylation.

The ability of our approach to predict methylated and unmethylated CGIs, was then directly compared to the results obtained from *EpiGRAPH *[32], which were tested on our HEP-based CGI methylation data and the methylation data used by the *EpiGRAPH *system (Table 10). In order to compare the methods we used a binary methylation classification system as described in the Materials and Methods section.

**Table 10:** Comparison of accuracy using binary methylation classification.

Methylation classification	Dataset	Methods	*Acc *[%]	*CC*
binary	HEP^1^	*EpiGRAPH *– SVM linear kernel*	84.90	0.657
binary	HEP^1^	*EpiGRAPH *– Decision tree C4.5*	75.80	0.462
binary	HEP^1^	*Matlab *– Decision tree**	90.08	0.743
binary	*EpiGRAPH *^2^	*EpiGRAPH *– SVM linear kernel*	85.20	0.658
binary	*EpiGRAPH *^2^	*EpiGRAPH *– Decision tree C4.5*	78.60	0.524
binary	*EpiGRAPH *^2^	*Matlab *– Decision tree**	91.67	0.775
four classes	HEP^3^	*Matlab *– Decision tree**	89.39	0.707

*Validation was performed using 10 repetitions of 10 fold cross-validation

** Validation was performed using 10 fold cross-validation

^1^HEP CGI data (using our attributes and binary methylation classes)

^2^*EpiGRAPH *methylation data (using the default *EpiGRAPH *sequence attributes and binary methylation classes)

^3^HEP CGI data (using our attributes and four methylation classes)

The average accuracy (*Acc*) and correlation coefficient (*CC*) were used to measure fitness.

Both datasets were classified using two methods, SVM and decision tree, the former one with two different implementations, *EpiGRAPH *C4.5 and the *Matlab *decision tree (CART, version R2007a). In all cases we used default parameters.

The results obtained using both datasets are very similar, independently of the classifier used. Thus, we suggest that our attribute set is capable of predicting methylated and nonmethylated CGIs with a high degree of accuracy even without sampling and method specific parameter optimization (Table 10).

Unexpectedly, a different implementation of a simpler method such as a decision tree, obtained a better accuracy and CC than the more complex SVM with the default setting parameters. In addition, this method produces interpretable rules that can be used for a better understanding of the data and easily extended to the use of multiple classes.

As shown in table 10 we are able to predict four different classes with accuracy close to that of the binary methylation prediction. The comparison with a more sophisticated classifier, suggests that the new information in terms of CGIs attributes and tissue-specific methylation classes are the key factors that improve the CGI classification instead of the classifier themselves (i.e. method bias) [57].

## Conclusion

The analysis of DNA methylation has been based primarily on the use of binary models which predict DNA sequences to be methylated or unmethylated. We have presented a profile-based approach that is able to define novel CGI methylation data relationships which not only separated between constitutively methylated and unmethylated CGIs but also identified CGIs showing a differential degree of methylation across tissues and cell-types or a lack of methylation exclusively in sperm. Our approach differs fundamentally from previous studies since it does not specify CGI classes *a priori*. Instead, it employs unsupervised data clustering methods for the detection of groups of CGIs sharing a common tissue-specific degree of methylation as well as similar attributes. These types of clustering methods avoid the potential biases of the limited CGI dataset available here since they do not require pre-determined classes in order to detect homogeneous groups within the data.

The functional CGI profiles discovered in this work bring new insights into the features associated with CGI methylation susceptibility, which included their evolutionary conservation, their significance, as well as the evolutionary evidence of prior methylation. Moreover, the usefulness of this information in building a simple classifier demonstrated that the ability to predict CGI methylation is mostly based on the biological information and the relationships uncovered between different sources of knowledge. This information can be exploited for the improvement and development of new tools able to detect not only constitutive or tissue-specific CGI methylation with equally high degrees of accuracy, but CGI functionality across the genome as well.

Contrary to previous studies, our method does not rely on *ad hoc *thresholds in order to determine if a CGI is constitutively methylated, unmethylated or shows a tissue-specific degree of methylation [25,31,32,43].

This yielded a series of novel, functional CGI profiles that allowed us to measure the extent of tissue-specific CGI methylation within the genic environment. We found that the different functional regions of genes were not equally affected by methylation. Furthermore, we were able to determine biological attributes that influence both the functionality and the methylation status of the CGIs, allowing us to use this knowledge for the computational prediction of their methylation.

In addition to the insights provided by our approach we demonstrate that the attribute set used is able to predict four methylation classes conserving the accuracy provided by leading binary methylation classification methods.

## Methods

### 1.1 Tissue-specific CGI methylation

The methylation data of the Human Epigenome Project (HEP) were used for the analysis of tissue-specific CGI methylation [31]. They specify 1.9 million CpG methylation values, from 2,524 sequences ("amplicons") across human chromosomes 6, 20 and 22. Methylation levels were measured in 12 different healthy tissues and cell types. Methylation values stemming from the same tissue and CpG were averaged and only unique CpGs, whose methylation status has been measured in at least one of the twelve tissues, were retained. The CGIs selected for this study were determined via the *CpGcluster *algorithm [38] and the degree of CGI methylation was then calculated by averaging all methylation values per CGI. This was done separately for each of the twelve tissues and only CGIs that had at least two tissues where over 70% of their CpGs were defined by a methylation value were then included in the database. In order to minimize the impact of missing methylation values during the detection of the CGI methylation profiles, a CGI was determined to have a degree of methylation of 0.5 if it was not defined in a particular tissue. This value indicates neither a high nor low degree of methylation and introduces the least amount of bias without having to limit the database to CGIs whose methylation status was known in all 12 tissues.

### 1.2 CGI biological attributes

Biological attributes belonging to the following categories were then used to characterize each of the CGIs in the database: (1) *CGI-specific attributes*: This category included the *p-value *calculated using the *CpGcluster *algorithm as a measure of CGI significance, the CpG Observed/Expected ratio (*O/E ratio*), the sequence content in Guanine and Cytosine (*G+C content*) [58] as well as the average distance between the CpGs of each CGI (*CpG-distance*), which is a measure of CpG spacing. Furthermore it included the standard deviation of the CpG distances (*SD*), calculated as:

where *N *is the number of CpGs in the CGI, χ_*i *_the distance between two consecutive CpGs and  is the average distance between neighboring CpGs of a CGI. Furthermore, the frequency of the 16 possible dinucleotides was measured (*Dinucleotide content*).

(2) *Repetitive sequences*: This category included both the number of repetitive elements intersecting with a CGI (*Repetitive elements*) and the fraction of a CGI covered by a repetitive element (*Repetitive content*). The human repetitive elements were identified using the *RepeatMasker *program [59].

(3) *Evolutionary conservation*: Conservation was measured via the fraction of each CGI overlapping with a *PhastCon *and the number of *PhastCon *elements per CGI. The *PhastCon *elements we used were highly conserved across 17 vertebrate genomes [60]. We obtained the "most conserved"*PhastCons *that demonstrate a log-odds conservation score of 100 or better via the UCSC Genome Browser [61].

(4) *Structural and physicochemical properties: *This category included the local sequence bending (*Bending*) and the macroscopic sequence curvature (*Curvature*), calculated using the *banana *algorithm from EMBOSS [62]. Furthermore it included the four attributes quantifying the number of DNA helix turns (*Turns*), the number of bases per turn (*Bases per turn*), the degree of DNA sequence twist (*Degree of twist*)and the base-pair stacking energy (*Stacking energy*), measured in kilocalories per mol, were calculated via the *btwisted *algorithm of the EMBOSS toolkit [62] and averaged over the length of the sequence. The stacking energy is measured in negative kcal/mol and the normalization was performed to between 0 and 1, values close to zero indicate that higher energy is needed to separate a region of double-stranded DNA.

The amount of DNA cleavage (*DNA cleavage*) indicates the solvent-accessible surface area of the DNA [63,64]. This information and was provided for each individual CGI by Thomas D. Tullius of the Department of Chemistry and Eric Bishop of the Program in Bioinformatics at the University of Boston. *DNA cleavage *was computed by averaging the single nucleotide cleavage values over the length of the CGI. The remaining attributes are based on a recent method described in Goni et al. [40] for calculating the six helical force-constants used to measure the average deformability of the CGI sequence: rotational parameters *twist*, *tilt *and *roll *(measured in kcal/mol degree^2^), translation-related parameters *shift*, *slide *and *rise *(measured in kcal/mol Å^2^) [65].

Prior to the profile searching, we performed a filtering of potentially uninformative CGI attributes via the Principal Components Analysis (PCA) method [66] using the *Spotfire*^® ^*DecisionSite*^® ^system [67]. The principal components that included 90% of the cumulative Eigenvalue were then chosen for further analysis. The contribution of each attribute to the principal components was analyzed via the eigenvector plots shown in Table 2 of the Supplementary data. CGI attributes that did not have a coefficient value greater than 0.1 or smaller than -0.1 in any of the selected principal components [28] were determined to be uninformative and removed from the database. Though the principal components themselves represent a reduction of the dimensionality of the data, meaning that they capture the same variability of the data but with fewer attributes, they were not used for any of the cluster analyses since neither the CGIs nor the actual values of the attributes that form part of the principle components are known.

### 1.3 Genes and pseudogenes

The CGIs were assigned to 7 classes based on their association with pseudogenes acquired from http://www.pseudogene.org/ and protein coding genes annotated in the AceDB [68] which summarizes all curated cDNA data from GenBank [69], dbEST [70] and the RefSeq [71]. This database was chosen because it provides a richer view of the human transcriptome, with three to five times more transcripts than the UCSC Known Genes, RefSeq or Ensembl annotations [68]. CGIs overlapping with a TSS or the promoter proximal region were defined as two separate classes (*TSS*, *Promoter*), since the TSS-overlapping CGIs are generally thought to have a higher G+C content and higher degree of CpG enrichment than non-TSS overlapping CGIs even if they are in the vicinity of the promoter [72]. In addition, we determined if a CGI was part of the 3'UTR (*3'UTR*) and separated purely intronic CGIs (*Intron*) from those overlapping partially or completely with a CDS on either strand (*CDS*).

### 1.4 Highly conserved non-coding elements (HCNEs)

HCNEs were downloaded from the *Ancora *database [49], a web resource that provides data and tools for exploring the genomic organization of HCNEs in multiple genomes http://ancora.genereg.net/. The HCNEs available from *Ancora *were identified from BLASTZ net whole-genome alignments of the human (hg18) and mouse (mm8) genomes and correspond to regions that have at least 70% identity over 50 alignment columns.

### 2. Profile analysis

#### 2.1 Data clustering

The two datasets containing the CGI methylation data and the CGI attribute data were analyzed separately using unsupervised cluster learning methods. The resulting clusters were later (section 2.2) used to define CGI profiles that connect coincident methylation and attribute clusters. Two distinct clustering methods were employed in this step: hierarchical clustering [27,28,73,74] and k-means clustering [73,75,76]. All functions are part of the *Matlab *Statistics Toolbox^® ^[77] if not indicated otherwise. The hierarchical clustering was performed using the Euclidean distance and the complete linkage approach. The number of clusters was calculated using inconsistency threshold (ICT) and coefficient [75] as validity indices [78]. The k-means clustering was performed using the Euclidean distance. To reduce the sensitivity of the algorithm to the initial random cluster centroids, each of the k-means runs was repeated ten times and the best solution was chosen. We used the silhouette method [79] to estimate the number of clusters. The potentially optimal number of k-means clusters was then chosen in order to maximize the average distance between silhouette means (ADSM).

#### 2.2. Combining clusters from independent sources of information into CGI profiles

The probability of intersection (*PI*) was used to determine the most significant intersections between the attribute and methylation data clusters. It is based on the hypergeometric distribution and is an adaptive measure that is sensitive to small sets of examples while retaining specificity with large datasets. The *PI *is more sensitive to relationships between smaller but highly similar groups than other measures that are based solely on the number of instances in the intersection [37,80]. This measure gives the chance probability of observing at least *p *candidates from a profile *V*_*i *_within another profile *V*_*j *_[37] as:

where *V*_*i *_represents a cluster of CGIs defining profile of size *h*, *V*_*j *_is a cluster of CGIs defining a profile of size *n*, *p *is the number of CGIs in the intersection between two clusters and *g *is the total number of CGIs in the database. The *PI *was computed using custom *Matlab*^® ^scripts.

#### 2.4. Summarizing profiles and labeling instances from the datasets

Identifying the right number of clusters is an unsolved problem [78,81]. As expected, different validity indices [78] as well as distinct clustering methods provide inconclusive results. Therefore, we selected more than one option (2.1) and instead of optimizing typicality inter and intra clustering measurements we optimized the posterior probability of matching between two different sources of clusters (2.3). This process generated several redundant profiles originating from redundant clusters. To remove this redundancy, we re-clustered the centroids [34] of the profile methylation classes and obtained a reduced set of classes including *constitutive unmethylated*, *constitutive methylated*, *unmethylated in sperm*, and *differentially methylated*. Then, we replaced the original classes in the profiles by the reduced ones and used them to label each instance in the CGI sequence attribute database. In other words, we transformed an unsupervised problem into a supervised one [73].

#### 2.5. Selecting the relevant features from each profile

This transformation into a supervised problem (i.e., labeled data) allowed us to apply typical feature selection strategies to identify the most relevant attributes for each profile. We use the entropy as a discriminative measurement [73] implementing a decision tree [73,82] (CART, *Matlab *version R2007a) with default parameters. For each profile we use the labeled data covered by it. This process was locally carried out for each profile to identify which are the relevant features for a particular methylation pattern. This process could also be performed by a global decision tree including all profiles but with less interpretable results (i.e. very long rules).

#### 2.3. Predicting CGI methylation from profiles

The functional CGI methylation profiles were used to predict based on a classification tree with default parameters as described above, and labeled observations (2.4). The classifier performance was evaluated using 10-fold cross-validation (*crossvalind Matlab*, version R2007a), the accuracy (*Acc*), which represents the fraction of CGIs whose methylation profile was predicted correctly (equation 3) and the correlation coefficient (*CC*) on the test subset which combines both sensitivity and specificity (equation 4):

To compare results with other data sources we used another implementation of the decision tree in *EpiGRAPH *[32] and the support vector machine classifier from the same source. The *CC *was only used in the binary classification, where a CGI is classified as "methylated" if it was not constitutively unmethylated. Finally, we compensated the unbalanced number of observations per class in the non-binary experiments by oversampling the *unmethylated in sperm *and *differentially methylated *classes [57].

## Abbreviations

(ADSM): Average distance between silhouette means; (CART): Classification and regression tree; (CC): Correlation coefficient; (CD4): CD4 Tlymphocytes; (CD8): CD8 T lymphocytes; (CDS): Protein codingsequence; (CGI): CpG island; (DF): Dermal fibroblasts; (DK): Dermalkeratinocytes; (DM): Dermal melanocytes; (FL): Fetal liver; (FSM): Fetal skeletal muscle; (HM): Heart muscle; (ICT): Inconsistency threshold; (HCNE): Highly conserved non-coding element; (HEP): Human Epigenome Project; (LINE): Long interspersed nuclear Element; (MWW): Mann-Whitney-Wilcoxon test; (O/E ratio):Observed/Expected ratio; (PBC): Profile based class; (PCA): Principlecomponent analysis; (PI): Probability of intersection; (SD): Standard deviation of CpGdistances; (SINE): Short interspaced nuclear element; (SM): Skeletal muscle; (T-DRM): Tissue-specific differentially methylated region; (TSS): Transcription start site; (UTR): Untranslated region.

## Authors' contributions

CP created the database. CP and OH performed the experiments. CP, IZ and CV analyzed the data. CP and CV wrote the manuscript. IZ and CV conceived, designed the study and financed the experiments. CV supervised the project. All authors read and approved the final manuscript.

## Supplementary Material

Additional file 1 **CGI dataset**. This Excel table contains the CGI dataset that formed the foundation for our data-mining approach, as well as the classification of each CGI in the binary and tissue-specific methylation classes. Click here for file

Additional file 2 **Results of the Principle Component Analysis**. This Excel table contains the Eigenvector plots of the PCA that were used to measure the contribution of each attribute to the principal components as well as the principle components themselves. Click here for file

Additional file 3 **Table of significant cluster intersections**. This table shows the significant cluster intersections, ordered by size (*#CGIs*) and significance of each intersecting cluster (*PI*). Each cluster is identified by the data used in the clustering (**A**ttribute or **M**ethylation data) followed by the overall number of clusters, the clustering method (**H**ierarchical or **K**-means clustering) and the particular cluster used. Click here for file

Additional file 4 **Decision tree**. This Figure shows the decision tree used to predict the four CGI methylation classes. Click here for file

Additional file 5 **Distribution of HCNE-overlapping PBCs over the gene association classes**. Absolute number and percentage of CGIs in each PBC and gene-association class that overlap with a HCNE. A total of 3 conflicting CGIs that were determined to contain CDS but were part of a HCNE were excluded. Significant enrichment (*p*-value < 0.05) of methylation classes with HCNEs is marked bold and was determined via the Fisher exact test in conjunction with Bonferroni correction for multiple testing. Click here for file

Additional file 6 **Table of variable correlation**. Correlation analysis of all attributes shows that each relevant attribute was not replaceable by any other. Click here for file
