# Supplementary material for: Profile analysis and prediction of tissue-specific CpG island methylation classes
Source: BMC Bioinformatics. 2009 Apr 21;10:116. doi: 10.1186/1471-2105-10-116 (PMC2683815; doi:10.1186/1471-2105-10-116)
Supplement: Additional file 3 — Table of significant cluster intersections. This table shows the significant cluster intersections, ordered by size (#CGIs) and significance of each intersecting cluster (PI). Each cluster is identified by the data used in the clustering (Attribute or Methylation data) followed by the overall number of clusters, the clustering method (Hierarchical or K-means clustering) and the particular cluster used. [file 1471-2105-10-116-S3.doc]

**Additional file 3– Table of Significant cluster intersections**

This table shows the significant cluster intersections, ordered by size (*# CGIs*) and significance of each intersecting cluster (*P-value*). Each cluster is identified by the data used in the clustering (**A**ttribute or **M**ethylation data) followed by the overall number of clusters, the clustering method (**H**ierarchical or **K**-means clustering) and the particular cluster used.

| Intersecting clusters | # CGIs | P-value |
| --- | --- | --- |
| A4K2 ∩ M4K3 | 162 | 1.30E |
| A4K2 ∩ M3K2 | 162 | 3.52E |
| A4K1 ∩ M9H6 | 123 | 3.52E |
| A4K1 ∩ M4K3 | 109 | 3.25E |
| A4K1 ∩ M3K2 | 109 | 4.81E |
| A8H6 ∩ M9H6 | 87 | 4.81E |
| A8H8 ∩ M9H6 | 84 | 1.86E |
| A9H4 ∩ M3K1 | 81 | 1.77E |
| A8H3 ∩ M3K1 | 81 | 9.69E |
| A8H6 ∩ M4K3 | 80 | 9.69E |
| A8H6 ∩ M3K2 | 80 | 1.30E |
| A9H9 ∩ M4K3 | 74 | 1.30E |
| A8H8 ∩ M3K2 | 74 | 2.14E |
| A8H8 ∩ M4K3 | 74 | 2.14E |
| A9H9 ∩ M3K2 | 74 | 2.14E |
| A4K3 ∩ M3K1 | 71 | 2.14E |
| A9H2 ∩ M9H6 | 63 | 1.00E |
| A8H1 ∩ M9H6 | 63 | 1.86E |
| A9H4 ∩ M4K4 | 61 | 1.86E |
| A8H3 ∩ M4H4 | 61 | 2.87E |
| A8H3 ∩ M9H8 | 55 | 2.87E |
| A9H4 ∩ M9H8 | 55 | 1.59E |
| A4K3 ∩ M4K4 | 54 | 1.59E |
| A9H2 ∩ M4K3 | 53 | 2.49E |
| A8H1 ∩ M3K2 | 53 | 2.04E |
| A9H2 ∩ M3K2 | 53 | 2.04E |
| A8H1 ∩ M4K3 | 53 | 2.04E |
| A4K3 ∩ M9H8 | 48 | 2.04E |
| A9H7 ∩ M3K2 | 48 | 1.33E |
| A9H7 ∩ M4K3 | 48 | 2.06E |
| A9H1 ∩ M9H6 | 36 | 2.06E |
| A9H1 ∩ M4K3 | 32 | 1.11E |
| A9H1 ∩ M3K2 | 32 | 6.73E |
| A9H4 ∩ M4K2 | 24 | 6.73E |
| A8H3 ∩ M4K2 | 24 | 1.99E |
| A8H2 ∩ M9H6 | 23 | 1.99E |
| A9H3 ∩ M9H6 | 23 | 5.27E |
| A4K3 ∩ M4K2 | 21 | 5.27E |
| A9H3 ∩ M4K3 | 20 | 1.48E |
| A8H2 ∩ M4K3 | 20 | 4.95E |
| A8H2 ∩ M3K2 | 20 | 4.95E |
| A9H3 ∩ M3K2 | 20 | 4.95E |
| A9H4 ∩ M9H9 | 17 | 4.95E |
| A4K3 ∩ M9H9 | 15 | 2.49E |
| A4K3 ∩ M9H1 | 8 | 2.71E |
| A4K4 ∩ M4H1 | 5 | 1.89E |
| A4K4 ∩ M3K3 | 5 | 1.73E |
| A4K3 ∩ M9H4 | 5 | 2.61E |
| A8H6 ∩ M9H3 | 4 | 4.83E |
| A8H5 ∩ M4K1 | 2 | 4.01E |
| A9H6 ∩ M4K1 | 2 | 3.33E |
| A9H6 ∩ M3K3 | 2 | 3.33E |
| A8H5 ∩ M3K3 | 2 | 4.05E |
| A9H6 ∩ M9H5 | 1 | 4.05E |
| A8H5 ∩ M9H5 | 1 | 1.73E |
|  | | |
