# Supplementary figures and images for: Profile analysis and prediction of tissue-specific CpG island methylation classes
Source: BMC Bioinformatics. 2009 Apr 21;10:116. doi: 10.1186/1471-2105-10-116 (PMC2683815; doi:10.1186/1471-2105-10-116)

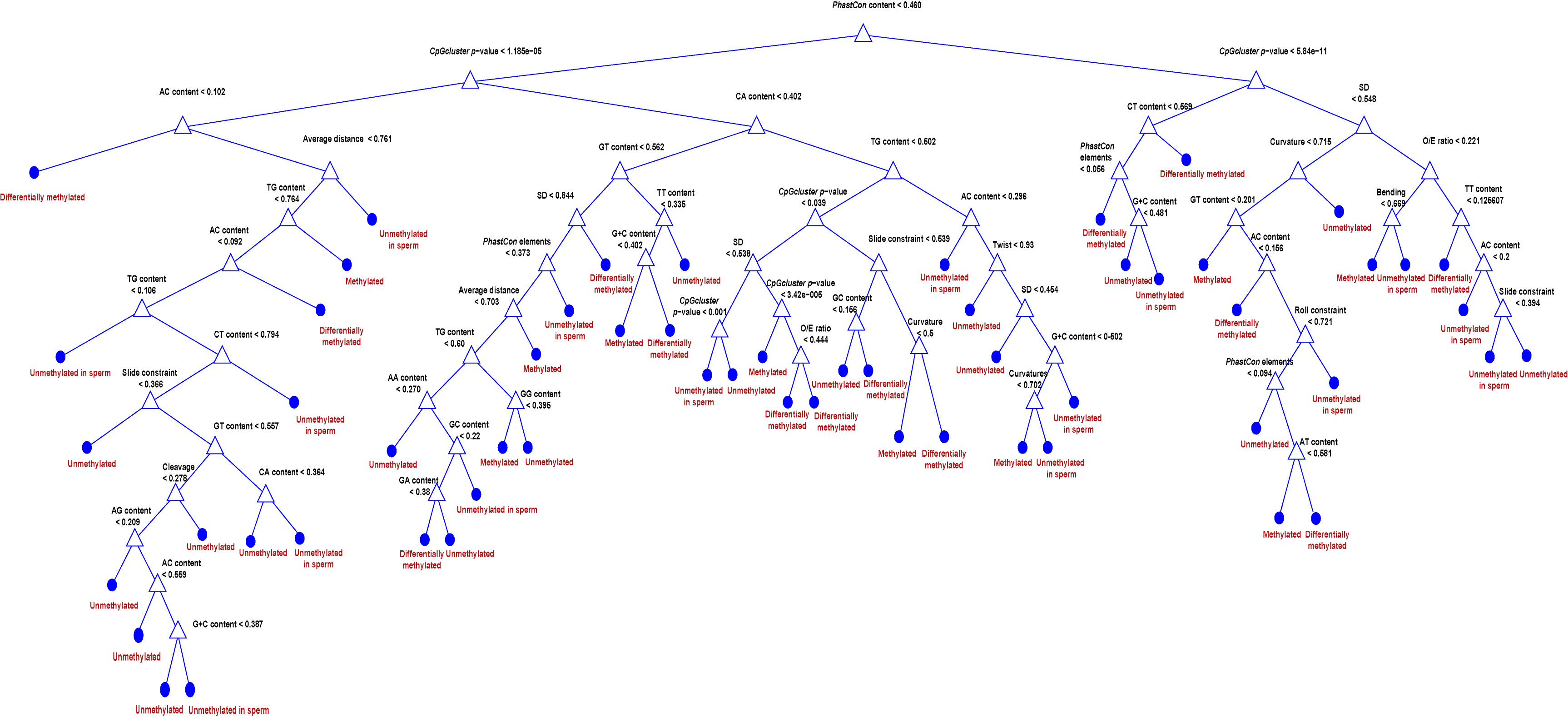

Supplement: Additional file 4 — Decision tree. This Figure shows the decision tree used to predict the four CGI methylation classes. [file 1471-2105-10-116-S4.png]
