# Supplementary material for: Profile analysis and prediction of tissue-specific CpG island methylation classes
Source: BMC Bioinformatics. 2009 Apr 21;10:116. doi: 10.1186/1471-2105-10-116 (PMC2683815; doi:10.1186/1471-2105-10-116)
Supplement: Additional file 5 — Distribution of HCNE-overlapping PBCs over the gene association classes. Absolute number and percentage of CGIs in each PBC and gene-association class that overlap with a HCNE. A total of 3 conflicting CGIs that were determined to contain CDS but were part of a HCNE were excluded. Significant enrichment (p-value < 0.05) of methylation classes with HCNEs is marked bold and was determined via the Fisher exact test in conjunction with Bonferroni correction for multiple testing. [file 1471-2105-10-116-S5.doc]

**Table 10 – Distribution of HCNE-overlapping PBCs over the gene association classes**

Absolute number and percentage of CGIs in each PBC and gene-association class that overlap with a HCNE. A total of 3 conflicting CGIs that were determined to contain CDS but were part of a HCNE were excluded. Significant enrichment (*p*-value < 0.05) of methylation classes with HCNEs is marked bold and was determined via the Fisher exact test in conjunction with Bonferroni correction for multiple testing.

| Gene association class | | Constitutively unmethylated (%) | Constitutively methylated (%) | Unmethylated in sperm (%) | Differentially methylated (%) |
| --- | --- | --- | --- | --- | --- |
| HCNEs | TSS | 2 (0.62) | - | - | - |
|  | Promoter | 9 (2.79) | 3 (3.57) | - | 1 (2.27) |
|  | 3’UTR | 1 (0.31) | - | - | 1 (2.27) |
|  | CDS | - | - | - | - |
|  | Intron | 2 (0.62) | 3 (3.57) | - | 2 (4.55) |
|  | Pseudogene | 1 (0.31) | - | - | - |
|  | NA | 1 (0.31) | - | 1 (2.27) | 3 (6.82) |
|  | Total # of conserved CGIs that are HCNEs | 16 (4.95) | 6 (7.14) | 1 (2.27) | **7 (15.92)** |
| HCNEs not overlapping  *PhastCons* | TSS | 1 (0.31) | - | - | - |
|  | Promoter | 1 (0.31) | - | - | - |
|  | 3’UTR | - | - | - | - |
|  | CDS | - | - | - | - |
|  | Intron | - | - | - | 2 (4.55) |
|  | Pseudogene | - | - | - | - |
|  | NA | - | 1 (1.19) | - | - |
|  | Total # of not conserved CGIs that are HCNEs | 2 (0.62) | 1 (1.19) | - | 2 (4.55) |
| Total # CGIs per PBC | | 323 | 84 | 44 | 44 |
